# Ursodeoxycholic acid inhibits pneumonia caused by PRCV through the activation of TLR4-IRF3 mediated type Ⅰ interferon pathway

**DOI:** 10.1186/s13567-026-01721-1

**Published:** 2026-03-02

**Authors:** Xingcui Zhang, Guisong Liao, Jinman Ding, Zhiwei Sun, Yi Zhong, Yanwen Song, Yi Li, Zhenhui Song

**Affiliations:** College of Veterinary Medicine, Southwest University, Chongqing, China

**Keywords:** Ursodeoxycholic acid, PRCV, TLR4-IRF3/JAK-STAT signaling pathway, antiviral mechanism

## Abstract

Porcine Respiratory Coronavirus (PRCV) is a genetic variant of Transmissible Gastroenteritis Virus (TGEV). It is only pathogenic to the respiratory tract, mainly manifesting as atypical interstitial pneumonia and mild subclinical symptoms. In addition, PRCV can also serve as a potential animal respiratory coronavirus model for the study of human respiratory coronaviruses. Developing effective PRCV antagonists is of great significance for the prevention and control of this disease. In this study, we found that Ursodeoxycholic acid (UDCA) can significantly inhibit the infection of PRCV in Porcine respiratory epithelial cells (NPTR). Further studies have shown that UDCA inhibits PRCV mainly through two mechanisms: First, UDCA can directly disrupt the components of the viral envelope and induce the disintegration of viral structure; Second, UDCA can significantly promote the secretion of IFN-β in NPTR cells, enhance the phosphorylation and nuclear translocation of STAT1, and up-regulate the expression of the interferon-stimulated genes ISG15 and MX1. Molecular dynamics simulations showed that UDCA can be embedded into the hydrophobic pocket of the TLR4 dimerization domain, thereby activating the TLR4-IRF3 signaling pathway, inducing the production of IFN-β, and inhibiting PRCV infection. Schaftoside, an inhibitor of the TLR4 signaling pathway, can effectively reverse the anti-PRCV activity of UDCA. Finally, an ex vivo lung tissue slice model of piglets was established to verify that UDCA can effectively reduce the PRCV viral load and inflammatory response in lung tissues. The results of this study provide a scientific basis for the development of antiviral drugs against PRCV and offer new insights into the research on human respiratory coronaviruses.

## Introduction

PRCV is an important member of the family *Coronaviridae* and the order *Nidovirales*, and taxonomically belongs to the genus *Alphacoronavirus* [1]. The genomic homology between PRCV and TGEV is approximately 96%. This high similarity confers strong cross-protective immunity, whereby antibodies induced by PRCV infection can effectively neutralize TGEV [2]. PRCV is high transmissible and primarily targets the respiratory system. Its infection is mainly confined to the pulmonary compartment, which can induce pathological manifestations such as pulmonary consolidation and bronchointerstitial pneumonia [3]. The pathogenic mechanisms of PRCV bears profound similarities to that of human respiratory coronaviruses, making it a unique model system for investigating human coronavirus infections [4].

PRCV exhibits predilection only in the respiratory tract, mainly as atypical interstitial pneumonia or mild subclinical symptoms. However, when PRCV is co-infected with other respiratory viruses (e.g., PRRS, SI) and other pathogenic microbes, can significantly exacerbate respiratory symptoms. This kind of coinfections can prolong the duration of systemic fever, reduce growth rates, and even may cause sudden death of infected pigs [5]. Therefore, it is of great significance to study the pathogenic mechanism of PRCV and develop effective antiviral drugs for the control and prevention of porcine respiratory coronavirus disease.

UDCA is a dihydroxy bile acid with unique physicochemical properties. Since it was first separated from bear bile in 1954, UDCA shown a variety pharmacological activities, including cytoprotective, antioxidant, immunomodulatory, bile-regulatory, anti-apoptotic, and antiviral effect [6–9]. Clinically, UDCA is clinically used mainly to dissolve cholesterol stones and treat liver and biliary diseases such as primary biliary cholangitis (PBC) [10]. At the cellular level, UDCA exerts its protective effects by regulating apoptosis and autophagy [11]. It inhibits the mitochondria-dependent apoptosis pathway, reducing apoptosis, while activating autophagy to clear damaged organelles and proteins, maintaining cellular homeostasis [12]. UDCA can regulate the inflammatory response and immune function through a variety of signaling pathways, and has potential therapeutic potential in the treatment of intestinal inflammation [13–19]. Recent studies have demonstrated that UDCA can inhibit the membrane fusion process of coronaviruses such as SARS-CoV-2 and modulate host innate immune responses [20]. Extensive studies further show that UDCA has significant inhibitory effects on acute and chronic inflammatory responses induced by various etiologies, including physical, chemical, and bacterial factors [21]. Additionally, studies have demonstrated that UDCA can reduce intestinal inflammation and alleviate LPS-induced intestinal inflammatory damage by reducing the expression of pro-inflammatory cytokines [22]. In this study, we evaluated the effect of UDCA on PRCV infection at both cellular and animal model levels. In addition, we investigated the molecular mechanism which UDCA inhibits PRCV replication, aiming to provide new insights into the development of antiviral strategies against PRCV.

## Materials and methods

### Virus, cell and animals

The PRCV used in this study was maintained in the laboratory and stored in liquid nitrogen tanks. Porcine respiratory epithelial cells (NPTR) were cultured in DMEM medium supplemented with 10% fetal bovine serum (FBS), and all cell cultures were maintained in a humidified incubator with 37 °C and 5% CO₂. Virus propagation and titration were performed in NPTR cells. Viral titer was determined by 50% tissue culture infective dose (TCID_50_) assay using the Reed-Muench and was expressed as TCID_50_ per mL. The piglets used for lung slicing were purchased from the Experimental Animal Center of Southwest University. Piglets were maintained under specific pathogen-free (SPF) conditions and used at the age of 15 to 20 days. All animal experiments were approved by the Animal Ethics and Use Committee of Southwest University, Chongqing, China.

### Reagents and antibodies

The UDCA and Schaftoside used in this study were both provided by Med Chem Express, a life science reagent supplier. Antibodies in the experiments included a mouse monoclonal antibody against PRCV-N (purchased from Beijing Subenyuanhe Biotechnology Co., Ltd.), β-tubulin/GAPDH antibodies, a rabbit polyclonal antibody against ISG15, a mouse monoclonal antibody against GAPDH, a rabbit monoclonal antibody against tubulin, a mouse polyclonal antibody against STAT1, a rabbit polyclonal antibody against p-STAT1, a rabbit recombinant antibody against TLR3, a rabbit polyclonal antibody against TLR4, a mouse monoclonal antibody against IRF3, and a rabbit polyclonal antibody against p-IRF3. All aforementioned antibodies were purchased from Proteintech Group, Wuhan. A rabbit polyclonal antibody against MX1 was purchased from Boster Biological Technology Co., Ltd., Shanghai.

### Cell viability

When the confluence of the cells in culture wells reached 85%–90%, the administration of the drug was initiated. A 100-μL aliquot of diluted UDCA solution was added to each well, and then the plate was incubated for 24 h in a cell culture incubator at 37 °C. After incubation, 10 μL of CCK-8 solution was added to each well, and then incubated 1 h. The absorbance was measured at 450 nm using a microplate reader to assess cell viability. The formula for calculating the percentage of cell viability is:

$$\text{Cell Viability}\hspace{0.17em}=\hspace{0.17em}[(\mathrm{As}-\mathrm{Ab})/(\mathrm{Ac}-\mathrm{Ab})]\hspace{0.17em}\times \hspace{0.17em}100\mathrm{\%},$$

where represents the absorbance of experimental wells (containing cells, medium, CCK-8, and drugs at different concentrations), Ac represents the absorbance of control wells (containing cells, medium, and CCK-8), and Ab represents the absorbance of the blank well (containing medium and CCK-8 without cells).

### Western blot

After the corresponding drugs or virus treatment of cells in 6-well plates, 200 μL of pre-cooled RIPA lysiscontaining protease inhibitor cocktail was added to each well to extract total cellular proteins, while RIPA lysis buffer containing phosphatase inhibitor cocktail was used to isolate phosphorylated proteins. Protein samples were subjected to 10% sodium dodecyl sulfate–polyacrylamide gel electrophoresis (SDS-PAGE) and the gels were excised guided by molecular weight markers, and the target protein bands were electrophoretically transferred to polyvinylidene fluoride (PVDF) membranes. The membrane was blocked with TBST solution containing 5% skim milk, bovine serum albumin (BSA) or quick-blocking buffer, then washed with TBST, and incubated with the corresponding primary antibodies at 4 °C overnight. After TBST washing, the membrane was incubated with secondary antibodies conjugated with horseradish peroxidase (HRP) at room temperature for 1 h. After another round of TBST washing, protein bands were detected using enhanced chemiluminescence (ECL) reagents. The relative expression level of target protein was quantified using ImageJ software, normalized to β-tubulin or GAPDH as loading control.

### Indirect immunofluorescence assay

NPTR cells were seeded on the coverslips of 24-well plates. After different treatments, the cells were fixed with 4% paraformaldehyde for 30 min and then permeabilized with 0.1% Triton X-100 at room temperature for 10 min. Subsequently, the cells were blocked with 5% bovine serum albumin (BSA) at room temperature for 1 h. After washing, primary antibodies (anti-PRCV-N, anti-TLR4, anti-IRF3, and anti-STAT1) were added and incubated at 4 °C overnight. After PBS washing, the cells were incubated with goat anti-mouse IgG H&L (Alexa Fluor 488) or Alexa Fluor 594-conjugated secondary antibodies at room temperature for 1 h in the dark. Nuclei were stained with DAPI for 5 min. Indirect immunofluorescence imaging of coverslip samples was performed using an inverted laser confocal microscope (LSM 800, ZEISS).

### Virus inactivation, attachment, internalization, replication and release assay

Direct Inactivation Group: PRCV at MOI = 0.1 was mixed with 200 μM UDCA at 37 °C for 3 h. The cells were pre-cooled at 4 °C for 1 h, and the medium was replaced with the UDCA-PRCV mixture, and incubated at 4 °C for 2 h. After washing with PBS for 3 times, the basic culture medium was added, and incubated at 37 °C for 36 h. The cell samples were collected and the viral gene copy number was measured.

Adsorption Assay Group: NPTR cells were pre-treated with 200 μM UDCA at 37 °C for 1 h, then treated with a mixture of UDCA-PRCV (MOI = 0.1) at 4 °C for 1 h, and the cell samples were collected to measure the viral gene copies.

Internalization Assay Group: NPTR cells were pre-cooled at 4 °C for 1 h and infected with PRCV (MOI = 0.1) at 4 °C for 1 h. Unbound viruses were removed by washing with cooled PBS, and the culture medium was replaced with DMEM containing UDCA (200 μM), and incubate at 37 °C for 2 h. The cell samples were collected to measure viral gene copy number.

Replication Assay Group: NPTR cells were infected with PRCV (MOI = 0.1) and incubated at 37 °C for 6 h. After removing the viral inoculum and washing three times with PBS, the medium was replaced with DMEM containing 200 μM UDCA, and cells were incubated at 37 °C for 10 h. The cell samples were collected to measure viral gene copy number.

Release Assay Group: NPTR cells were infected with PRCV at MOI = 0.1 at 37 °C for 16 h, then treated with 200 μM UDCA for 1 h. The cell supernatant was collected to measure viral gene copy number.

### Observation of virus particles by Electron microscopy

According to the manufacturer's instructions, the collected viral supernatant was concentrated using a virus concentration kit. For the UDCA-treated viral samples, 20 μL of the sample was dropped on a copper grid for negative staining. After 3 min, the excess liquid was absorbed with filter paper, and the grid was placed on 2% phosphotungstic acid staining for 1–2 min, and then blotted again. The morphology changes of the viral particles were observed using a transmission electron microscopy (TEM) (JEM-1400FLASH, JEOL, Japan), and the images were collected from the copper grids.

### Quantitative qPCR analysis

The RNA transcription levels of PRCV-N, IFN-β, TLR4, IL-6, TNF-α, ISG15, and MX1 were detected by quantitative reverse transcription (RT)-qPCR. Total RNA was extracted using TRIzol reagent according to the manufacturer’s protocol, and cDNA was synthesized using the PrimeScript RT Kit (TaKaRa, Japan). Subsequently, real-time quantitative PCR analysis was performed using 2 × Universal SYBR Green Fast qPCR Mix and gene-specific primers (Table 1). The relative mRNA expression levels were normalized to GAPDH as an endogenous reference gene and calculated using the 2^⁻ΔΔCt^ method. In addition, absolute qPCR quantification of the PRCV genomic copies/μL was performed using primers PRCV N -forward and N-reverse.

**Table 1:** **Primer sequences for quantitative qPCR**

Names				Sequences (5’ to 3’)
PRCV	F		TTCAACCCCATAACCCTCCAACAA	
	R		GGCCCTTCACCATGCGATAGC	
ISG 15	F		CCACGGCCATGGGTAGG	
	R		GATGCCATCATGCAGTCCCT	
MX 1	F		CAGAGGCAGCGGAATTGTG	
	R		GCTGTCCCGGTAACTGACTT	
IFN-β	F		AGCACTGGCTGGAATGAAACCG	
	R		CTCCAGGTCATCCATCTGCCCA	
TLR 4	F		CCGGGTCACTTCTGTTCACG	
	R		CTAATGTTAGGAACCACCTGCAC	
β-tubulin	F		CTCTTCCAGCCCTCCTTCC	
	R		GGTCCTTGCGGATGTCG	
GAPDH	F		GAAGGTCGGAGTGAACGGATTT	
	R		TGGGTGGAATCATACTGGAACA	
IL-6	F		ATCAGGAGACCTGCTTGATG	
	R		TGGTGGCTTTGTCTGGATTC	
TNF-α	F		ACTGCACTTCGAGGTTATCGG	
	R		GCTTTGACATTGGCTACAACG	

F: forward; R: reverse.

### Animal experiments: precision cut lung slice

Three-week-old piglets were anesthetized with 5% isoflurane and then injected intravenously with 10% KCl. The entire lung was rapidly excised, and one lung lobe was selected. The tracheal orifice was identified and a blunt-tipped long needle was slowly inserted. A 50-mL syringe was connected to aspirate 1.5% low-melting-point agarose pre-warmed in a 37 °C incubator, which was then slowly injected into the lung while withdrawing the needle until the entire lung lobe expanded. The tracheal opening was clamped, and the lobe was embedded in ice. Once the agarose had completely solidified, lobe of the lung was trimmed and sectioned at a single bronchus. The modified tissue block was sliced with a tissue slicer, and the slice thickness of 150–450 μm. The lung slices were immediately transferred to 24-well plate containing WME medium, incubated at 37 °C with 5% CO₂ for 3 h, and then the medium was changed to restore ATP levels and remove cellular debris. Medium was renewed every 24 h. After 24 h incubation, the PRCV was inoculated to the active PCLS with TCID_50_ = 1 × 10^5^. All animal experiments were approved by Southwestern University's Institutional Animal Care and Use Committee (Animal Protocol Approval Number: IACUC-20230509–02) and followed the National Institutes of Health's Animal Performance Guidelines.

### Paraffin sections with HE staining

Lung tissue specimens were fixed in 10% neutral-buffered formalin at room temperature for 24–48 h, then dehydrated through a gradient of ethanol (70%, 80%, 95%, 100%), cleared with xylene, and finally embedded in paraffin. The tissue was cut into sections of 4 μm thickness using a rotary microtome, attached to a sticky slide, and baked at 60 °C for 1 h. Following dewaxing in xylene and rehydration through a gradient of ethanol (100%, 95%, 80%, 70%), sections were stained with Harris hematoxylin for 8 min, washed with running water for 10 min, differentiated with 1% acidic ethanol for 5 s, stained with 0.2% ammonia water, and finally counterstained with eosin Y solution for 90 s. Finally, sections were dehydrated in turn by different concentrations of ethanol gradient (70%, 80%, 95%, 100%), then clarified with xylene, and finally sealed with neutral resin.

### Computer molecular docking simulation

Homology modeling of TLR3 and TLR4 proteins was performed using the Swiss-Model webserver. The three-dimensional structure of UDCA was downloaded from the PubChem database. Protein receptors and drug ligands were processed using AutoDock Tools 1.5.7 to remove water molecules, add polar hydrogens, and assign charges. Stereoisomers were generated and energy minimization was performed using Chem3D 22.0.0, followed by conversion to PDBQT format suitable for docking. The docking grid setting size for TLR3 was set with dimensions of 126 Å × 104 Å × 102 Å (x, y, z axes) centered at coordinates (6.111, 2.972, -8.684), while the TLR4 grid box was set to 94 Å × 96 Å × 88 Å centered at (10.722, 2.972, -3.0). These grid boxes were designed to encompass the active sites of the TLRs. The ligand (UDCA) was set as flexible, and the receptors (TLRs) were set as rigid during docking simulations using AutoDock Vina 1.1.2. The top 10 conformations were generated, and the highest-affinity conformation was visualized using PyMOL 2.5.

### Statistical analysis

All results in this study were statistically analyzed by GraphPad Prism 9.0 software using one-way analysis of variance (ANOVA) and t-test. By the general criteria of statistical field, the differences are considered to be statistically significant at *P* < 0.05, when *P* > 0.05, it is considered that there is no statistically significant difference between groups, by “ns”; when *P* < 0.05, there is a statistically significant difference between groups, indicated by (*); *P* < 0.01 indicates highly significant intergroup difference, indicated by (**); and *P* < 0.001 is an extremely significant difference, marked with (***). The number of biological replicates is n = 3.

## Results

### UDCA co-treatment and post-treatment modalities showed significant inhibition effects of PRCV infection in vitro

**Figure 1:**
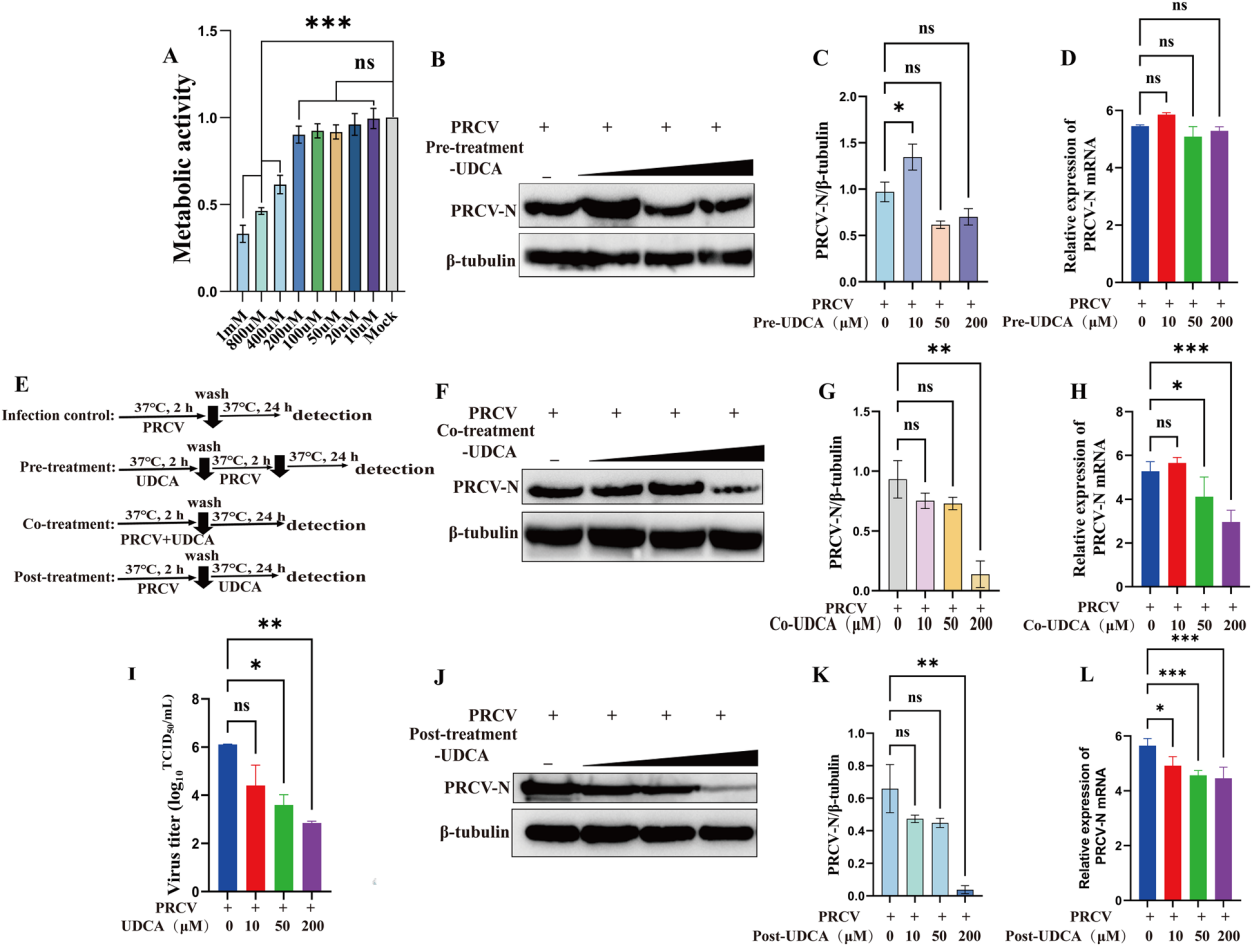
**Inhibitory effects of pre-treatment, co-treatment, and post-treatment with UDCA (10, 50, and 200 μM) on PRCV infection**. **A** Cell viability was measured by CCK-8 assay. **B**–**D** PRCV-N protein levels in the pre-treatment group were detected by western blotting with quantitative analysis via Image J, and PRCV-N nucleic acid levels were measured by RT-qPCR. **E** Schematic diagram of the experimental setup for UDCA and PRCV treatment in NPTR cells. **F**–**H** PRCV-N protein levels in the co-treatment group were detected by western blotting with quantitative analysis via Image J, and PRCV-N nucleic acid levels were measured by RT-qPCR. **I** Viral titers of post-treatment with UDCA (10, 50, and 200 μM) were determined by TCID_50_ assay. **J**–**L** PRCV-N protein levels in the post-treatment group were detected by western blotting with quantitative analysis via Image J, and PRCV-N nucleic acid levels were measured by RT-qPCR. The results are the averages ± SD of experiments performed in triplicate. Student’s *t* tests were used for statistical analyses. ns, no significance; *, *P* < 0.05; **, *P* < 0.01; ***, *P* < 0.001.

To evaluate the inhibitory effects of UDCA on PRCV in vitro, and to determine its optimal working concentration and treatment modality, NPTR cells were subjected to pre-treatment, co-treatment, and post-treatment with UDCA at 10 μM, 50 μM, and 200 μM, respectively, as detailed in Figure 1E. CCK-8 assays showed that UDCA at a concentrations of 200 μM and below had minimal impact on the viability of NPTR cell, maintaining above 80%, and was not significantly discrepancy from the control group (Figure 1A). Therefore, 200 μM was selected as the maximum concentration for subsequent experiments. The anti-PRCV activity of UDCA was evaluated by detecting expression level of PRCV-N protein and the number of nucleic acid copies in NPTR cells at 24 h post-infection. Experimental results showed that pre-treatment with 10 μM, 50 μM and 200 μM UDCA had no expected effect with western blot and RT-qPCR analysis (Figures 1 B to D), co-treatment and post-treatment with UDCA at 50 μM and 200 μM significantly reduced PRCV gene copy numbers compared to those of the untreated group (Figures 1H and L), with the post-treatment group exhibiting a concentration-dependent inhibitory effect. Western blot analysis further confirmed that PRCV-N protein expression was substantially decreased in both the co-treatment and post-treatment groups at 200 μM UDCA (Figures 1F, G, J and K), with a more stable efficacy in the post-treatment group. In addition, the TCID_50_ assay showed that PRCV infection was inhibited in a dose-dependent manner after UDCA treatment (Figure 1I). Collectively, these results support the selection of 200 μM UDCA post-treatment as the optimal condition for further experiments.

### UDCA post-treatment showed significant inhibition effects on PRCV infection at different time points in vitro

**Figure 2:**
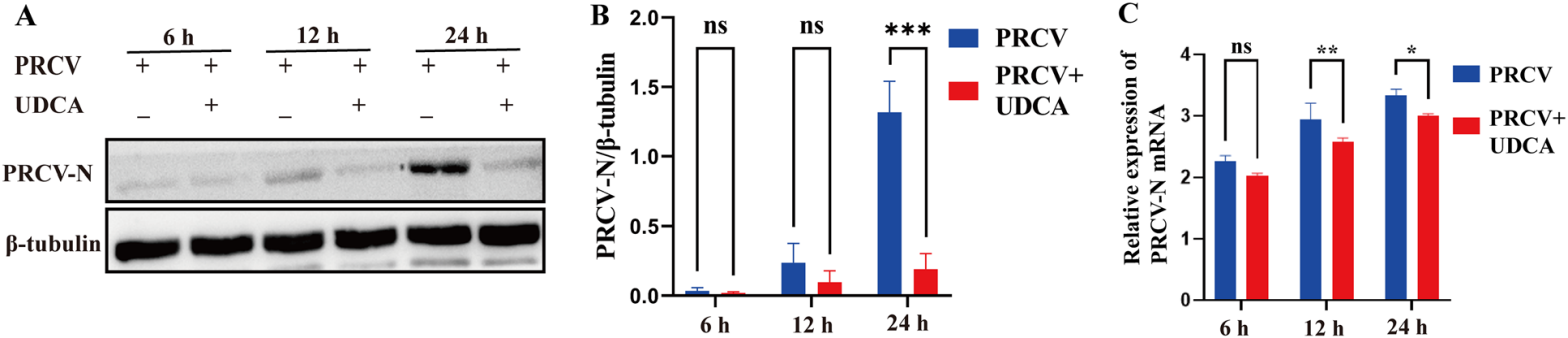
**Inhibitory effects of post-treatment with UDCA on PRCV infection at different time points in vitro**. **A**–**C** PRCV-N protein levels at different time points were detected by western blotting with quantitative analysis via ImageJ, and PRCV-N nucleic acid levels were measured by RT-qPCR. The results are the averages ± SD of experiments performed in triplicate. Student’s *t* tests were used for statistical analyses. ns, no significance; *, *P* < 0.05; **, *P* < 0.01; ***, *P* < 0.001.

To assess the inhibitory effect of post-treatment with 200 μM UDCA on PRCV infection, samples collected at different time points were analyzed by western blot, quantitative PCR (qPCR), and immunofluorescence assay (IFA). The results showed that PRCV-N protein expression was significantly decreased in the UDCA-treated group at 24 h post-PRCV infection and the decreased expression were also observed at other time points (Figure 2A), the corresponding grayscale analysis of western blot in Figure 2A was showed in Figure 2B. Viral nucleic acid quantification analysis showed that the viral gene copy number was reduced in the UDCA intervention group at both 12 and 24 h post-infection (hpi), and the inhibitory effect was more obvious at 12 h (Figure 2C). Collectively, these findings confirmed that UDCA effectively inhibited PRCV infection in vitro.

### UDCA inhibited the virus during the replication stage and destroyed the viral particle structure of PRCV

**Figure 3:**
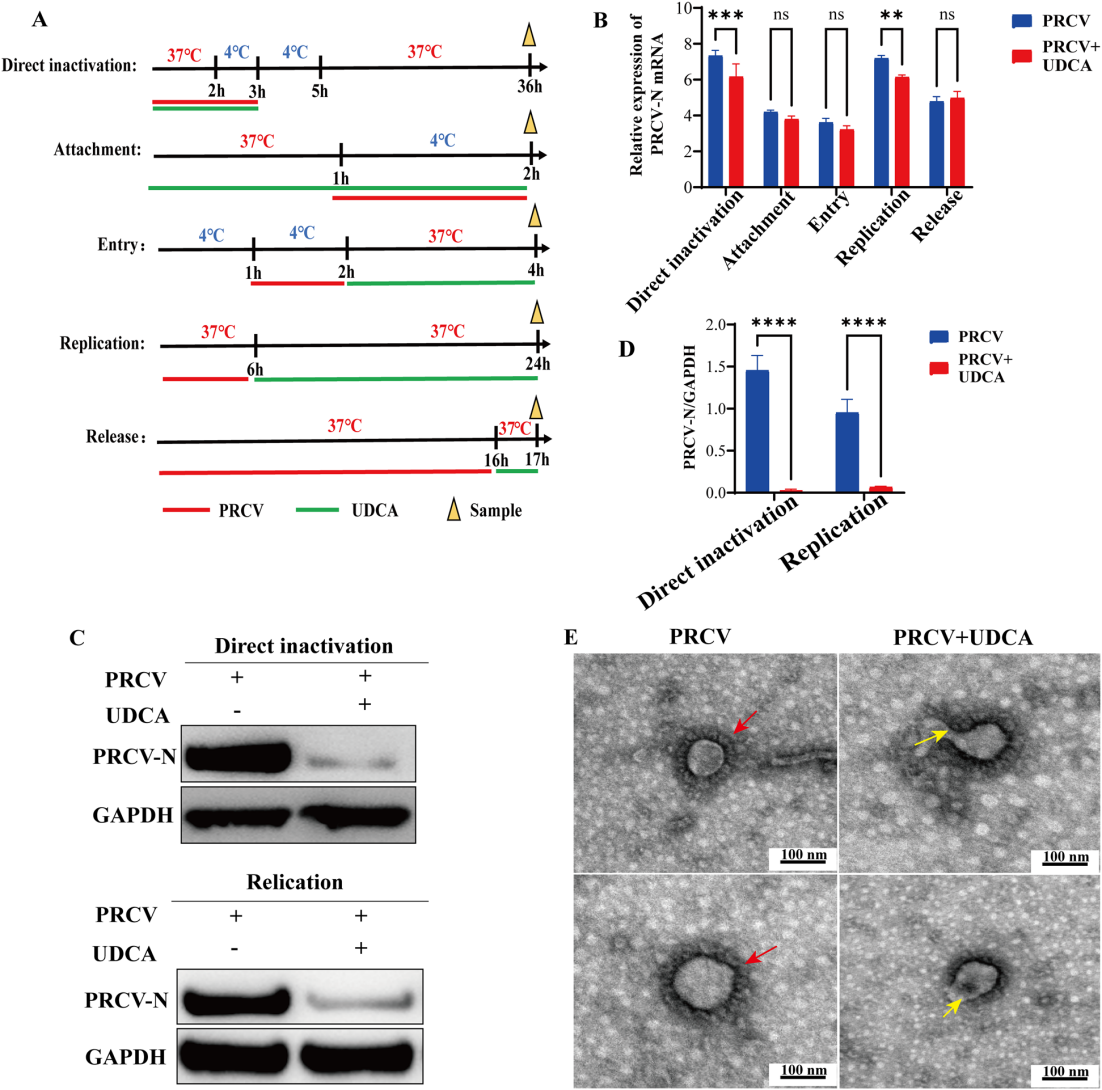
**Assessment of the phases of PRCV infection inhibited by UDCA**. **A** Schematic diagram of UDCA and PRCV treatment at different infection phases. **B** RT-qPCR analysis of PRCV inhibition by UDCA at different phases. **C**–**D** Western blot detection of PRCV-N protein levels during the direct inactivation and replication phases, with quantitative analysis via ImageJ. Additionally, purified PRCV virions were incubated at 37 °C for 3 h in the presence or absence of UDCA. Viral suspensions were then prepared for electron microscopy observation. Representative images of PRCV virions under different experimental conditions are shown. **E** Electron microscopy observation of the PRCV and the effect of UDCA on PRCV. Red arrows indicate intact viral particles, while yellow arrows denote disrupted virions.

To investigate the effects of UDCA on different stages of PRCV infection, this study established experimental groups including direct inactivation, adsorption treatment, internalization treatment, replication treatment, and release treatment, and samples were collected from each group (Figure 3A). Absolute RT-qPCR analysis of PRCV gene copy numbers in cells and supernatant showed that UDCA treatment significantly reduced PRCV-N gene copies in NPTR cells in both the direct inactivation and replication treatment groups (Figure 3B). Western blot analysis confirmed these findings, demonstrating that UDCA significantly reduced the expression of PRCV-N protein at both the direct inactivation and replication stages (Figure 3C), the Figure 3D was the corresponding grayscale. To further validate whether UDCA directly disrupted the structure of PRCV virions, concentrated virus suspensions were subjected to phosphotungstic acid negative staining and observed under a transmission electron microscopy (TEM). The results showed that PRCV viral particles exhibited intact spherical morphology with a diameter of approximately 100 nm, consistent with characteristic features of typical coronavirus particles, in contrast, the structural integrity of viral particles in the PRCV + UDCA treatment group was compromised (Figure 3E). These observations indicate that UDCA has the ability to directly disrupt the structure of PRCV viral particles, thereby impairing their effectiveness in infecting host cells. Overall, the findings of this study demonstrate that UDCA not only directly disrupts the structure of PRCV viral particles but also effectively neutralizes PRCV viral particles.

### UDCA processing is closely related to the cellular innate immune signaling pathways through transcriptomic data

**Figure 4:**
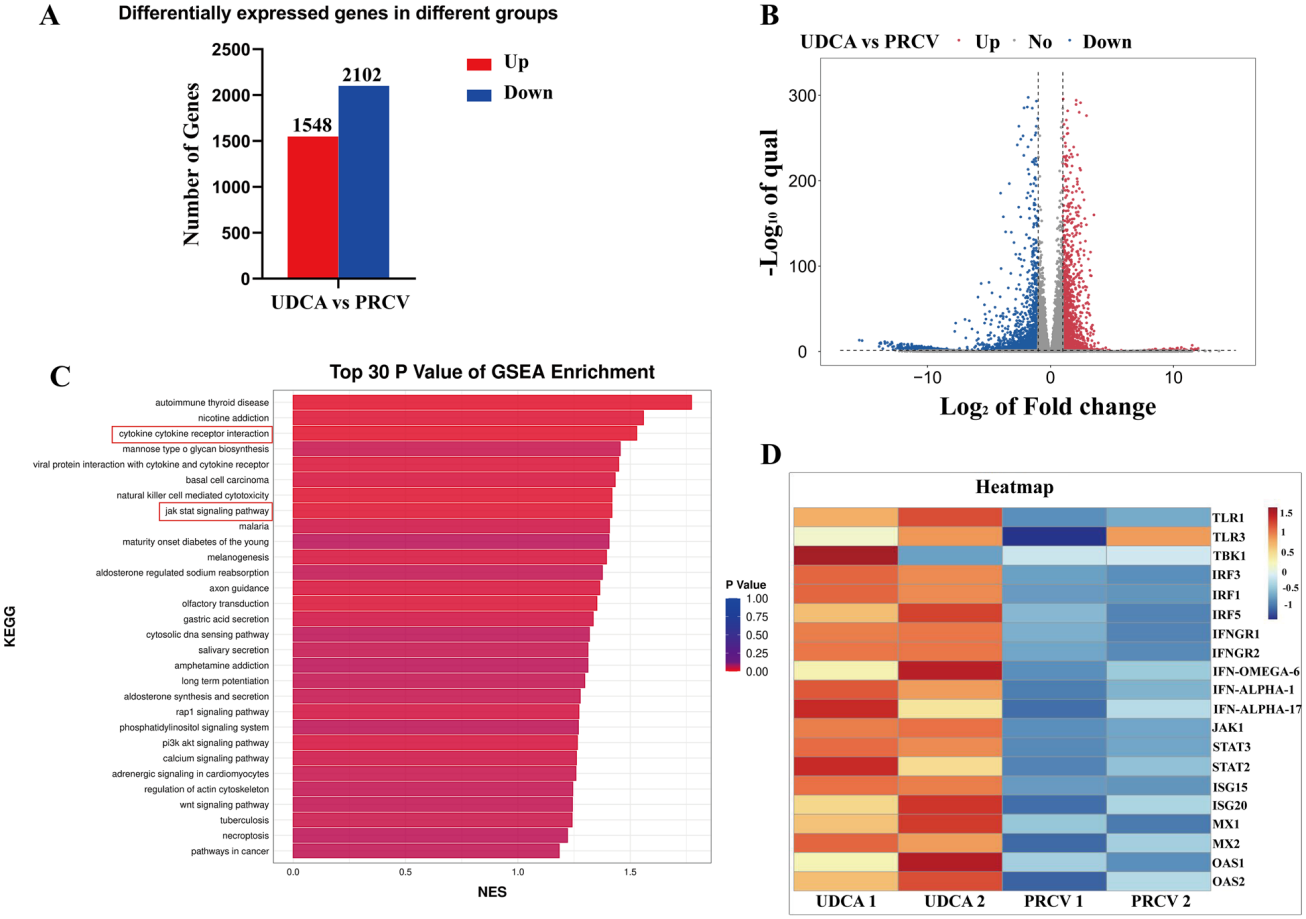
**Identification of signaling pathways associated with UDCA treatment and PRCV infection based on transcriptomic data**. **A** Bar chart of differentially expressed genes. **B** Volcano plot of differentially expressed genes. **C** Gene Set Enrichment Analysis (GSEA) plot. **D** Correlation cluster marker heat map.

By comparing the gene expression profiles of the UDCA-treatment group and the PRCV-infected group, a total of 1548 significantly upregulated genes and 2102 significantly down-regulated genes were identified (Figure 4A). Integrated analysis of differentially expressed genes. Gene Set Enrichment Analysis (GSEA) plot (Figure 4C) revealed significant associations between JAK-STAT signaling pathways under UDCA treatment. The volcano plot (Figure 4B) showed that UDCA treatment significantly upregulated the expression of antiviral genes MX1 and ISG15. The relevant cluster marker heat map (Figure 4D) further confirmed the close association of these genes with the JAK-STAT signaling pathways under UDCA treated.

### UDCA significantly activated IFN-β signaling and antiviral gene expression

**Figure 5:**
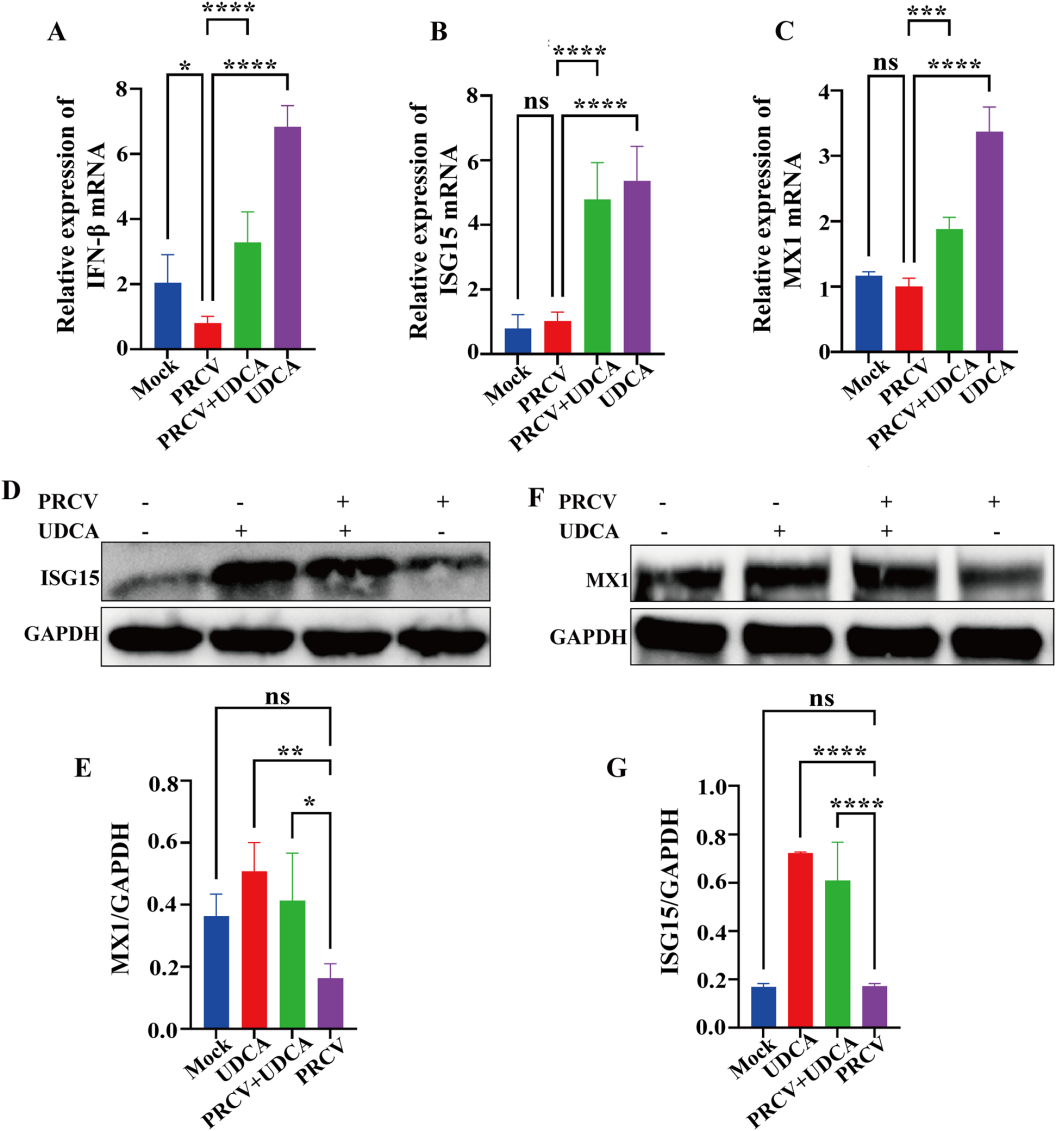
**Regulatory effects of UDCA intervention and PRCV infection on IFN-β transcriptional levels and protein/nucleic acid levels of interferon-stimulated genes (ISGs) in NPTR cells**. **A** Changes in IFN-β secretion levels in NPTR cells following UDCA treatment and PRCV infection. **B**–**C** Effects of UDCA treatment and PRCV infection on transcriptional levels of ISG15 and MX1. **D**–**G** Effects of UDCA treatment and PRCV infection on protein levels of ISG15 and MX1, with quantitative analysis performed via ImageJ. The results are the averages ± SD of experiments performed in triplicate. Student’s *t* tests were used for statistical analyses. ns, no significance; *, *P* < 0.05; **, *P* < 0.01; ***, *P* < 0.001.

Compared with the control group, the expression level of IFN-β was significantly decreased in the PRCV-infected group, while the expression level of IFN-β significantly increased in the UDCA treatment group. An increasing trend of IFN-β expression was observed in the post-PRCV-infection + UDCA-treated group (Figure 5A). These findings suggest that PRCV infection suppresses cell IFN-β secretion to facilitate viral replication, while UDCA treatment stimulates IFN-β secretion in NPTR cells, thereby counteracting PRCV-mediated inhibition and enhancing immune defense. RT-qPCR analysis showed that the transcription levels of ISG15 and MX1 were significantly higher in the UDCA-treated group compared to those of the PRCV-infected group (Figures 5B and C). Western blot results corroborated these findings, indicating that ISG15 protein expression was markedly upregulated in both the UDCA-treated and post-PRCV-infection + UDCA-treated groups, and MX1 protein expression was also significantly elevated (Figures 5D to G). Overall, the JAK-STAT signaling pathway in the UDCA-treated NPTR cell model were consistent with the transcriptomic predictions, validating the effectiveness of the model the accuracy of the transcriptomic data.

### UDCA significantly activated STAT1 phosphorylation and promoted its nuclear translocation

**Figure 6:**
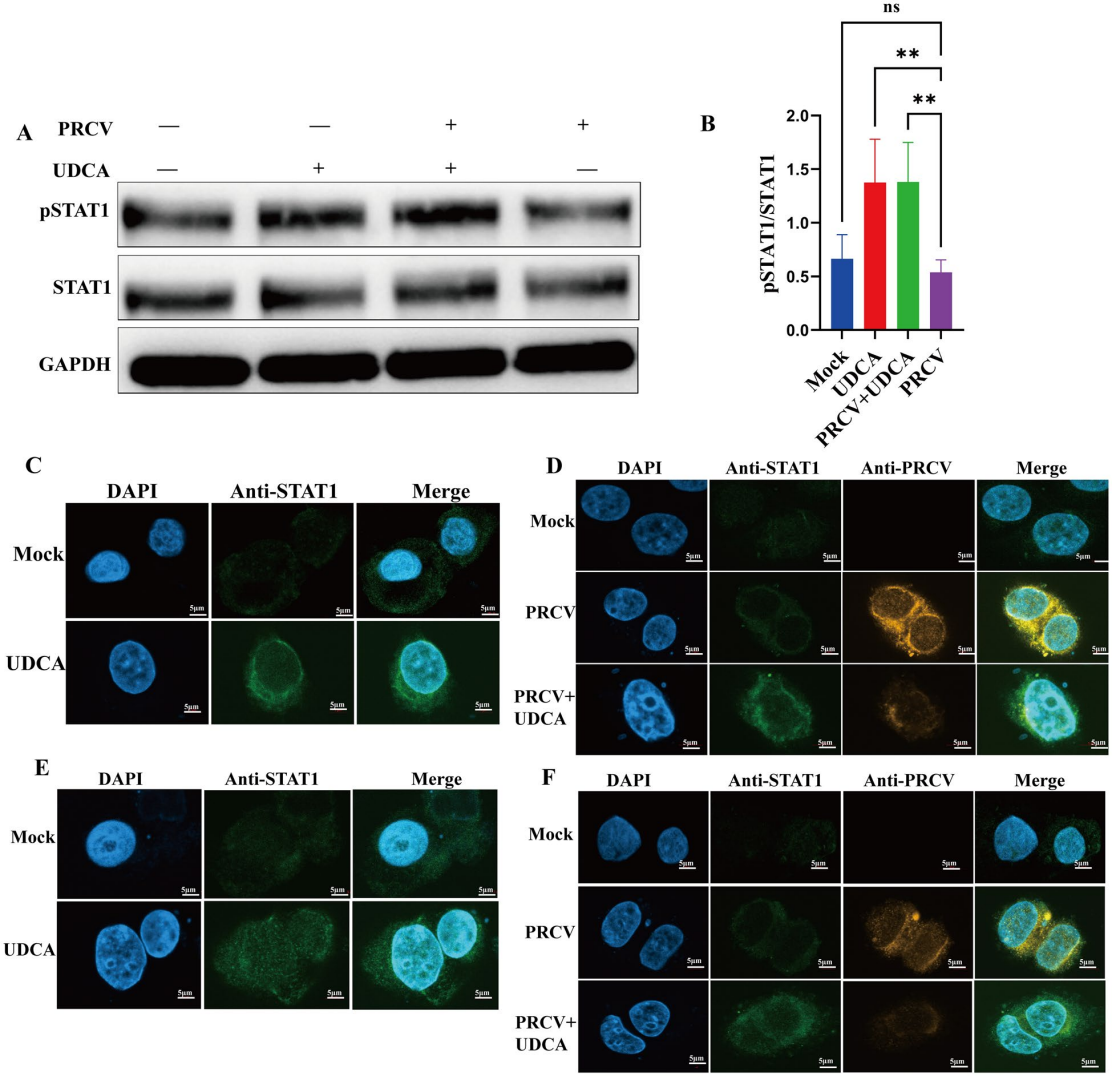
**Regulatory effects of UDCA treatment and PRCV infection on STAT1 phosphorylation and nuclear translocation**. **A**–**B** Changes in p-STAT1/STAT1 protein levels following UDCA treatment and PRCV infection, with quantitative analysis performed via ImageJ. **C**–**D** Dynamic regulatory effects of 12 h UDCA treatment on STAT1 nuclear translocation. **E**–**F** Dynamic regulatory effects of 24 h UDCA treatment on STAT1 nuclear translocation.

In the physiological state, STAT1 exists in an unphosphorylated form in the cytoplasm. When the pathogen invades, the cells stimulate STAT1 nuclear translocation, while the virus usually disrupts the JAK-STAT signaling pathway by inhibiting the tyrosine phosphorylation and nuclear translocation of STAT1, so as to escape from the host immune surveillance. To validate whether UDCA enhanced antiviral immunity by activating this pathway, the present study used western blot to detect the expression of p-STAT1 and STAT1 proteins in cells and utilized IFA to analyze STAT1 nuclear translocation after UDCA treatment. The results showed that the ratio of p-STAT1/STAT1 was slightly decreased in the PRCV-infection group compared with the control group, but the change not statistically significant. In contrast, the levels of STAT1 phosphorylation were significantly higher in both the UDCA-treated group and the post-PRCV-infection UDCA-treated group (Figure 6A), quantitative analysis of the western blot was performed via ImageJ (Figure 6B). IFA results showed that fluorescence intensity of STAT1in the cell nucleus was significantly enhanced at both 12 h (Figure 6C and 6D) and 24 h (Figure 6E and 6F) after UDCA treatment. Collectively, these findings indicate that UDCA enhances the host antiviral immune response against PRCV infection by promoting IFN-β secretion, activating the JAK-STAT signaling pathway, enhancing STAT1 phosphorylation and nuclear translocation, resulting in increasing gene and protein expression of antiviral cytokines (ISG15 and MX1).

### TLR4-IRF3 signaling cascade mediated UDCA-induced IFN-β activation

**Figure 7:**
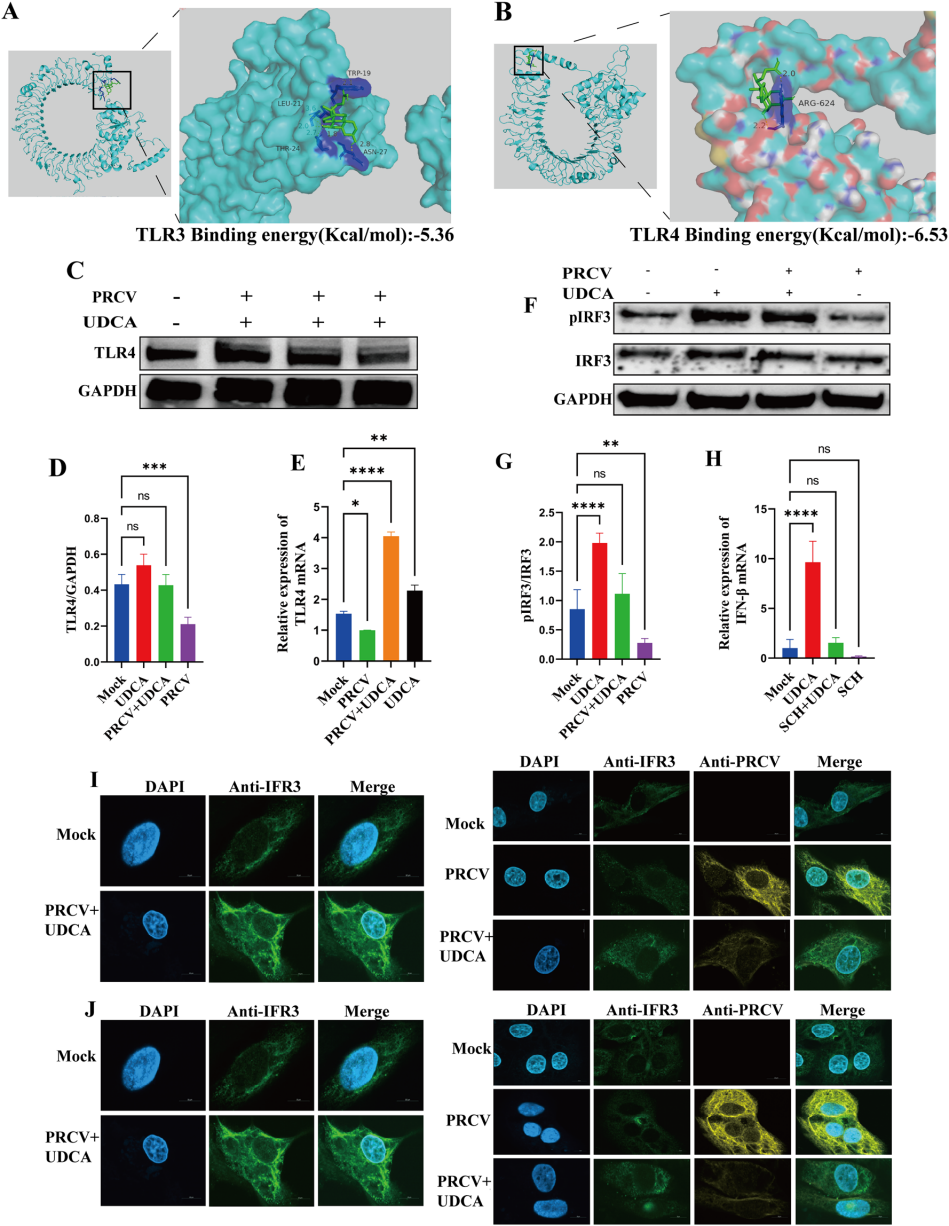
**Impact of UDCA treatment and PRCV infection on the upstream TLR4-IRF3 signaling pathway governing IFN-β production**. **A**-**B** Molecular docking of UDCA with Toll-like receptor 3 (TLR3) and Toll-like receptor 4 (TLR4) proteins. (Panel B depicts UDCA stably embedded within the TLR4 hydrophobic binding pocket). **C** TLR4 protein expression levels; **D** Representative immunoblot (optional, if shown); **E** TLR4 transcriptional (mRNA) levels. **F**-**G** Alterations in phosphorylated IRF3 (p-IRF3) to total IRF3 protein ratio following UDCA treatment. **I**-**L** Temporal dynamics of UDCA-mediated regulation of IRF3 nuclear translocation assessed at 12 h and 24 h post-treatment. **H** Modulation of IFN-β secretion levels in NPTR cells subsequent to treatment with the TLR4 inhibitor Schaftoside.

In the host innate immune system, Toll-like receptors 3 and 4 (TLR3/TLR4) recognize pathogen-associated molecular patterns (PAMPs), which then activate interferon regulatory factor 3 (IRF3) and promote the production of type I interferons (IFN-I). Results of Molecular docking simulations revealed that UDCA had the lower binding free energy (-6.53 kJ/mol) for TLR4 (Figure 7B) compaired to that (-5.36 kJ/mol) of the TLR3 (Figure 7A). Complementary molecular dynamics simulations showed that UDCA stably embedded in the hydrophobic binding pocket of the TLR4 dimerization structural domain, forming variety of intermolecular interactions, including hydrogen bonds and van der Waals forces. These computational results suggest that UDCA may induce TLR4 dimerization by targeting this domain, thereby initiating downstream signaling, and ultimately enhancing IFN-β expression. Under basal conditions, IRF3 exists in the cytoplasm in a non-phosphorylated state. Pathogen invasion typically triggers the phosphorylation of IRF3 and its subsequent nuclear translocation. Notably, PRCV infection suppresses this key process. Consistent with this observation, western blot and IFA results demonstrated that PRCV infection significantly diminished phosphorylated IRF3 (p-IRF3) levels, whereas UDCA treatment enhanced IRF3 phosphorylation and facilitated its nuclear translocation (Figures 7 F, G, I, J). This indicates that UDCA effectively antagonized PRCV-mediated suppression of IRF3 activation, ultimately promoting IFN-I transcription. PRCV infection markedly downregulated TLR4 mRNA expression (Figure 7E). However, UDCA treatment restored the TLR4 transcription level to baseline levels. Furthermore, TLR4 expression was increased in the UDCA-treated group, but this increase did not reach statistical significance. However, PRCV infection caused a pronounced reduction in TLR4 protein (Figures 7C and D), revealing that UDCA reverses PRCV-induced suppression of TLR4. To delineate the functional role of the TLR4 pathway in UDCA-induced IFN-β expression, the cells were treated with the specific TLR4 inhibitor, Schaftoside, alongside UDCA. Subsequent cellular RNA quantitative RT-qPCR analysis was performed to assess the changes in IFN-βlevels. The results confirmed that UDCA significantly upregulated IFN-β transcription (Figure 7H), which was completely abolished by co-treatment with Schaftoside. This critical experiment validates that the TLR4 signaling cascade constitutes the essential molecular target by activating IRF3 and inducing IFN-β expression by UDCA.

**Figure 8:**
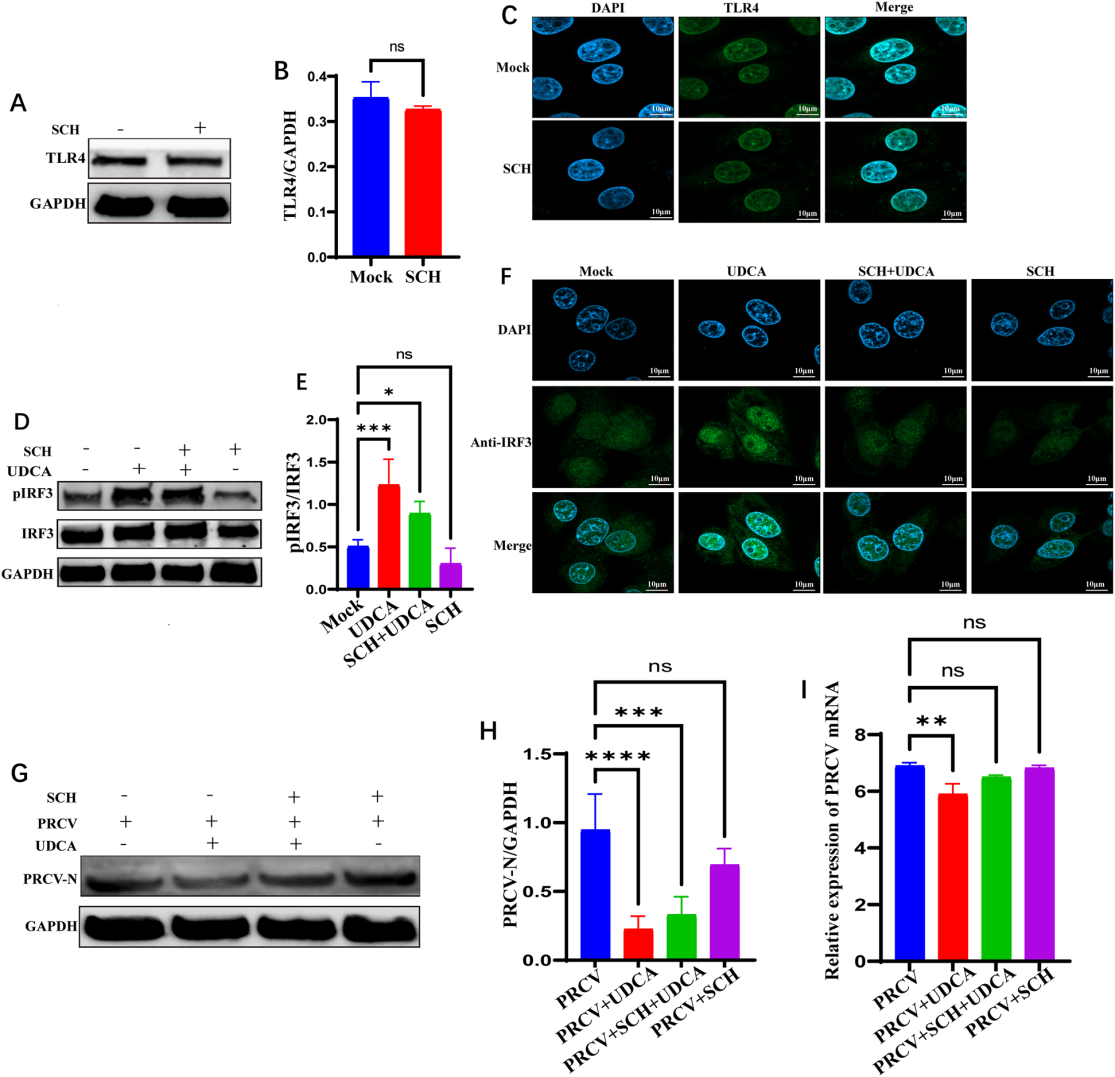
**Effects of Schaftoside and UDCA treatment on the TLR4-IRF3 signaling pathway upstream of IFN-β**. **A**–**C** Effects of Schaftoside treatment on TLR4 protein expression levels and fluorescence intensity. **D**–**F** Changes in p-IRF3/IRF3 protein levels and nuclear translocation following Schaftoside treatment. **G**–**I** Effects of Schaftoside treatment on PRCV protein expression levels and gene transcription levels.

The effects of The TLR4 signaling pathway inhibitor Schaftoside on TLR4 protein were investigated using western blot and IFA. The results of western blot showed that the expression of TLR4 protein was slightly decreased after the treatment of Schaftoside, but the change was not statistically significant (Figures 8A and B). IFA analysis also revealed that Schaftoside treatment did not significantly change the fluorescence intensity of TLR4 in the cytoplasm (Figure 8C), suggesting that Schaftoside may exert immunomodulatory effects by inhibiting TLR4 downstream signaling rather than acting directly on the TLR4 protein. The effect of Schaftoside and UDCA treatment on IRF3 activation was further analyzed by western blot and IFA. The results showed that Schaftoside monotherapy reduced the level of IRF3 phosphorylation, while UDCA treatment significantly increased the level of IRF3 phosphorylation. Conversely, treatment with Schaftoside and UDCA together attenuated UDCA-induced IRF3 phosphorylation (Figures 8D-E). IFA results further confirmed that UDCA treatment significantly increased nuclear IRF3 fluorescence intensity, whereas Schaftoside treatment attenuated this effect (Figure 8F). These findings indicated that UDCA activates IFN-β transcription by promoting IRF3 phosphorylation and nuclear translocation, while Schaftoside antagonizes this effect, suggesting the TLR4-IRF3 pathway could be a key target of their interaction. To further clarify the impact of UDCA and Schaftoside on the replication of PRCV, absolute RT-qPCR and western blot were executed to quantify PRCV-N gene copies and protein expression. Compared with the PRCV-infected group, UDCA treatment significantly reduced viral RNA copies and protein expression (Figures 8G–I). However, PRCV gene copies and protein expression were restored upon Schaftoside addition, indicating that Schaftoside, as a specific antagonist of the TLR4 signaling pathway, could effectively reverse the antiviral effects of UDCA. This result further validates the mechanism by which UDCA inhibits PRCV replication through activation of the TLR4-IRF3-IFN-β signaling axis.

### UDCA inhibited PRCV-induced inflammation and PRCV replication in precision-cut lung slices

**Figure 9:**
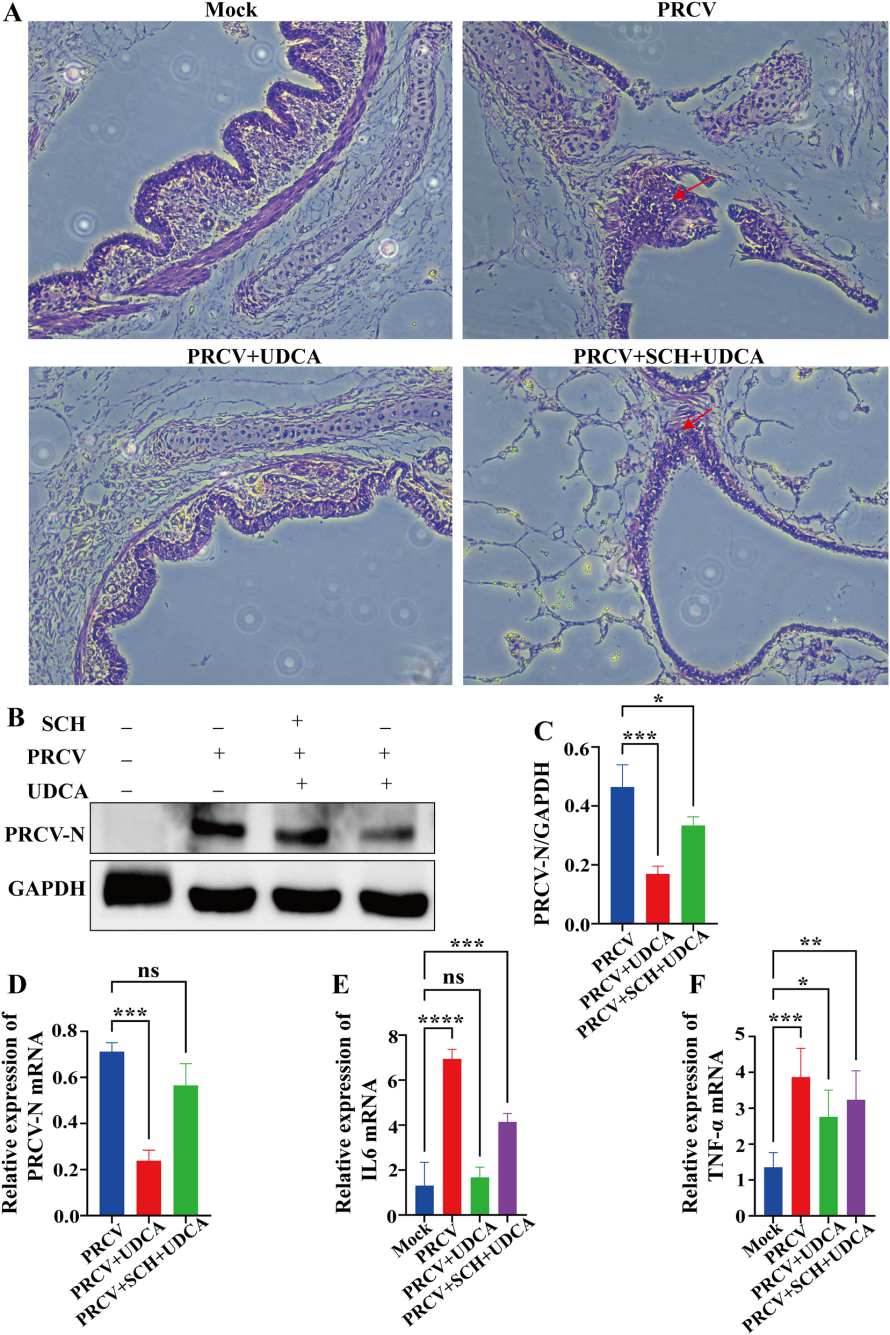
**Effects of Schaftoside and UDCA treatment on inflammatory factors and viral infection in organoid models**. **A** Effects of Schaftoside and UDCA treatment on inflammatory infiltration in PCLS. **B**–**D** Effects of Schaftoside and UDCA treatment on PRCV protein expression and gene transcription levels in PCLS. **E**–**F** Effects of Schaftoside and UDCA treatment on gene transcription levels of IL-6 and TNF-α in PCLS.

To investigate the impact of PRCV infection on the inflammatory cytokine expression in NPTR cells, hematoxylin and eosin (HE) staining was performed on four groups of PCLS after paraffin embedding in this study, respectively: PRCV Infection group, PRCV + UDCA co-treatment group, PRCV + UDCA + Schaftoside co-treatment group and Negative control group. Histopathological assessment was performed via HE staining, focusing on inflammatory cell infiltration, and it was found that there was a large amount of lymphocytic infiltration in the PRCV infection group, concomitant with significant disruption of the ciliary structure of the bronchus. While residual inflammatory cell infiltration could be observed in the groups that received combined treatment of Schaftoside and UDCA post-PRCV infection, while there was almost no such infiltration in both the negative control group and the treated solely with UDCA post-infection group. Furthermore, the bronchial ciliary structure of the bronchus in the latter two groups was maintained intact (Figure 9A). Subsequent quantitative RT-qPCR analysis of IL-6 and TNF-α gene expression levels in the sample groups showed a marked upregulation of both cytokines in the PRCV infection group relative to the negative controls. Notably, UDCA treatment significantly attenuated the PRCV-induced inflammatory response. Crucially, the addition of Schaftoside abrogated this anti-inflammatory effect, greatly diminishing UDCA's ability to suppress inflammation (Figures 9E, F). These molecular findings corroborated the histopathological observations of HE staining. To further elucidate the effect of UDCA and Schaftoside treatment on the replication of PRCV, this study quantitatively analyzed the viral gene copy number and protein expression levels by RT-qPCR and western blot analysis, respectively. The results showed that UDCA treatment significantly reduced both PRCV RNA copy number and viral protein expression compared to that of the PRCV infection group alone. However, the introduction of Schaftoside into the UDCA treatment regimen resulted in a discernible resurgence in viral gene copy and protein expression levels (Figures 9B-D). This key observation confirmed that Schaftoside, as a specific antagonist of TLR4 signaling pathway, could effectively counteract the antiviral effects of UDCA. Moreover, the mechanism of UDCA in orchestrating the TLR4-IRF3 signaling cascade activation to suppress PRCV replication was further validated using corroborative experiments utilizing an ex vivo organoid model.

## Discussion

PRCV is a highly contagious viral pathogen that affects the respiratory tract of pigs. It is widely exhibits in pig populations of all ages, manifesting a spectrum of respiratory symptoms including coughing, dyspnea, and labored breathing. Infection further leads to anorexia, weight loss, and pulmonary inflammation [23, 24]. As a natural mutant strain of TGEV, PRCV possesses broad host tropism and efficient transmission capabilities. Its tendency to co-infect with diverse porcine viral diseases exacerbates clinical manifestations and may induce immunosuppression, further impairing the protective efficacy of conventional vaccines [25]. PRCV has striking similarities with human respiratory coronaviruses SARS-CoV-2 in terms of pathogenesis and clinical manifestations, making it prototypical model system for the study of human respiratory coronaviruses [26]. Consequently, developing novel and effective therapeutic interventions is imperative for controlling emerging severe coronavirus diseases in both swine and human.

Recent studies have shown that UDCA may inhibit the entry SARS-CoV-2 into cells through direct interaction with host cell membranes. Furthermore, UDCA significantly downregulates the expression angiotensin-converting enzyme 2 (ACE2) by inhibiting Farnesoid X Receptor (FXR). This reduction in ACE2 availability impairs viral receptor binding, thereby diminishing the viral adhesion to host cells and subsequent internalization [27, 28]. While traditionally used as a therapeutic agent for liver and biliary diseases, UDCA has recently emerged as a compound with potential broad-spectrum antiviral activity. Compelling evidence indicates that its inhibition of FXR may be a mechanism by which it reduces the abundance of the ACE2 receptor, thus attenuating the susceptibility of target cells to SARS-CoV-2 invasion. Notably, FXR exerts an inhibitory effect on the transcriptional activity of interferon regulatory factor 3 (IRF3) through direct protein–protein interaction. This suppression inhibits the activation of downstream type I interferon signaling and suppresses the expression of antiviral ISGs [29–31]. Consequently, UDCA-mediated inhibition of FXR may facilitate the dissociation of the FXR-IRF3 complex, enhance the recovery of type I interferon signaling cascades and enhancing the transcriptional output of ISGs. This mechanistic suggests that UDCA has dual immunomodulatory roles in counteracting viral evasion strategies.

This study further confirms that UDCA can potently antagonize PRCV infection, a member of the same *Coronaviridae* family. In the NPTR cell model, the sequential pharmacological paradigms (pre-treatment, co-incubation, post-treatment) was adopted, and the results showed that UDCA significantly inhibited PRCV under co-incubation and post-treatment conditions. Subsequent mechanistic validation revealed that UDCA exerted its antiviral activity primarily at the stage of direct exting the virus and viral replicative phases (Figure 3A-D). Ultrastructural analysis by electron microscopy confirmed that UDCA directly impairs integrity of the viral envelope, impairing the stability of viral particle structure, thereby diminishing the infectivity of the virus in host cells. This virucidal effect is related to the intrinsic bile acid properties of UDCA, which facilitate hydrophobic interactions with the viral envelope lipid bilayer, thereby altering membrane fluidity and inducing structural disintegration. Moreover, the efficacy of UDCA in the PRCV infection model suggested an additional immunomodulatory mechanism: regulating the viral replication phase by activating antiviral signaling cascades, which ultimately promotes viral component clearance or inhibits the viral transcriptional processes and viral protein synthesis. Interferons (IFNs) constitute pivotal mediators of the host innate immune response and acts as broad-spectrum antiviral agents that influence cellular proliferation, orchestrate immunomodulation, and inhibits viral replication by the activation of diverse antiviral proteins. However, it is worth noting that several porcine coronaviruses including PRCV adopt multifaceted strategies to disrupt type I IFN signaling compromising STAT1 nuclear translocation, thereby inhibiting the activation of the JAK-STAT pathway, which in turn suppresses the antiviral function of ISGs [32, 33]. This dynamic evolutionary interplay between virus and host underscores the complexity of immunoregulatory networks. While prior findings of this study have confirmed the significant inhibitory effect of UDCA on PRCV infection, its molecular basis of antiviral action remains incompletely defined. Transcriptomics analysis subsequently identified a significant association between UDCA treatment, PRCV infection, and the interleukin-cytokine receptor interactions and JAK-STAT signaling pathways. Gene Set Enrichment Analysis (GSEA) revealed that differentially expressed genes (DEGs) distinguishing UDCA-treated from PRCV-infected groups were predominantly enriched in three pivotal regulatory tiers of the JAK-STAT pathway: type I/III IFNs (IFN-I/III) and its homologous receptor genes were significantly upregulated, and the expression of antiviral effector genes ISG15 and MX1 was increased. Experimental validation confirmed these findings: UDCA treatment significantly increased IFN-β secretion, enhanced STAT1 phosphorylation and nuclear translocation, and markedly upregulated both transcriptional levels and protein expression of downstream ISGs in NPTR cells. Together, these results demonstrate that UDCA activates the JAK-STAT signaling axis by enhancing the production of IFN-βand the propagation downstream signals.

The major antiviral mechanisms identified in this study are as follows: UDCA directly interacts with PRCV, destroying the structure of viral particles, thereby impairing viral invasion. It enhances the host antiviral IFN-β response via the TLR4-IRF3 pathway. Additionally, UDCA activates the JAK-STAT signaling pathway, promoting the expression of ISGs to inhibit PRCV replication.

**Figure 10:**
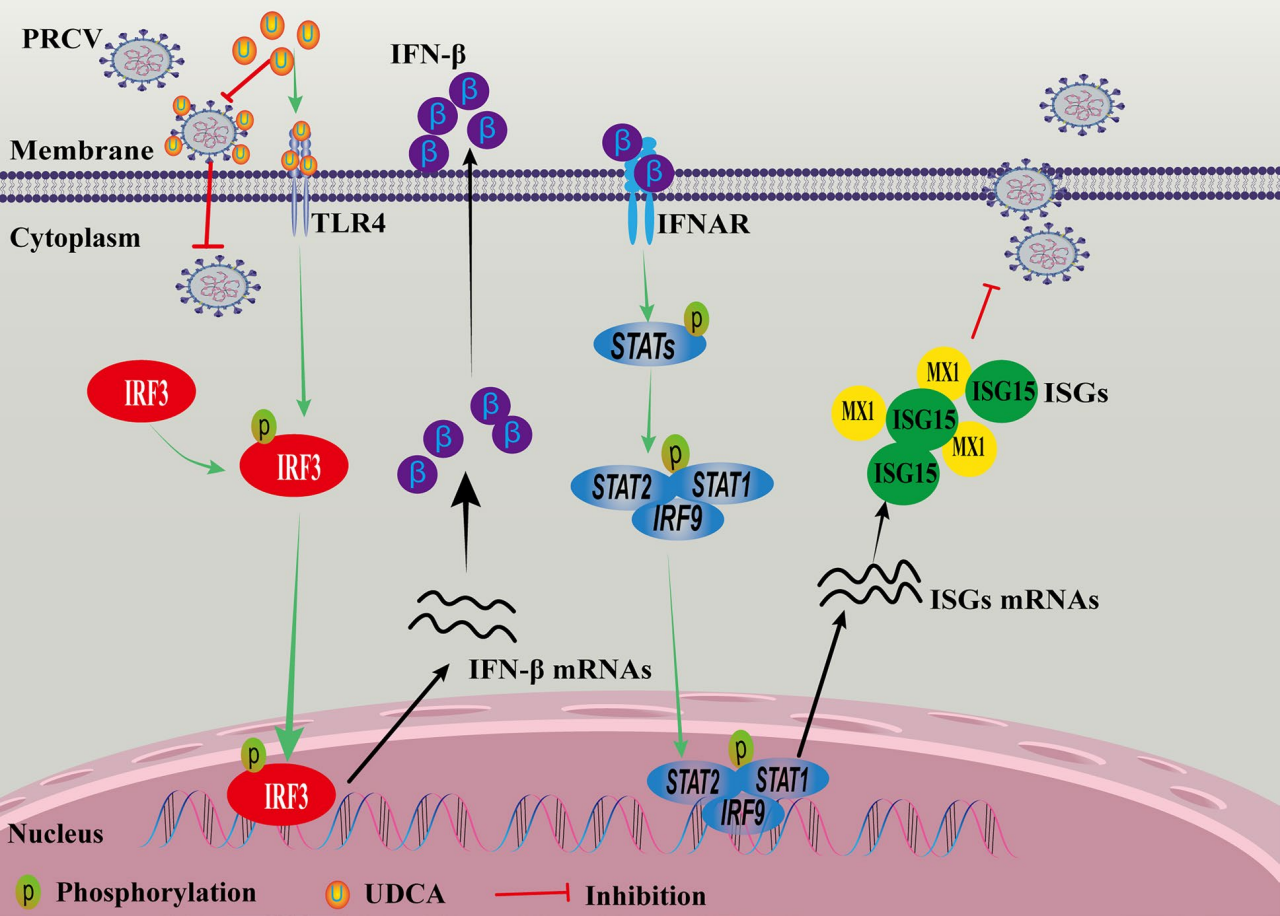
**The mechanism diagram of anti-PRCV effect of UDCA.**

In order to further elucidate the molecular mechanism of UDCA inhibiting PRCV replication through enhancing the secretion IFN-β, activating the JAK-STAT signaling pathway, increasing transcription of ISGs, boosting host innate immunity, antagonizing the innate immune suppression mediated by PRCV-mediated, the molecular docking experiments revealed that UDCA exhibits low binding energy with the TLR4 receptor, indicating a specific binding tendency. Further studies showed that UDCA promoted IRF3 phosphorylation and nuclear translocation, thereby activating IFN-β secretion. Inhibition the TLR4-IRF3 signaling pathway using the TLR4 receptor inhibitor Schaftoside significantly diminished UDCA-induced IFN-β levels, and restored PRCV protein expression and gene transcription. These findings indicate that UDCA activates downstream IRF3 phosphorylation and promotes IFN-β secretion via binding to TLR4 receptor. Furthermore, considering that the FXR is a core regulator of bile acid metabolism, its inhibition may enhance antiviral effects through two pathways: reducing the membrane localization of the viral receptor ACE2 and relieving negative regulation of interferon signaling pathways. UDCA may form a dual-mechanism “double insurance” activation mode of interferon pathway through the positive activation of TLR4 and the negative regulation of FXR.Overall, during the infection process of PRCV, the virus suppresses the innate immunity through multiple ways, especially by obstructing the production of IFNs and ISGs, thereby producing an immunosuppressive microenvironment. The intervention of UDCA destroyed this balance via dual mechanisms (Figure 10): first, after binding to TLR4, UDCA activates the TLR4-IRF3-IFN-β-JAK-STAT signaling cascade, thereby reshaping the viral inhibitory innate immune response; second, UDCA exerts a direct antiviral effect through its physicochemical properties. This discovery provides not only a novel strategic framework for the prevention and control of PRCV but also important insights for antiviral drug research against other highly variable coronaviruses.

## Data Availability

No datasets were generated or analysed during the current study.
